# Oral contraceptives and hypertension in women: results of the enrolment phase of Tabari Cohort Study

**DOI:** 10.1186/s12905-021-01376-4

**Published:** 2021-05-28

**Authors:** Mahdi Afshari, Reza Alizadeh-Navaei, Mahmood Moosazadeh

**Affiliations:** 1 Pediatric Gastroenterology and Hepatology Research Center, Zabol University of Medical Sciences, Zabol, Iran; 2 Gastrointestinal Cancer Research Center, Non-communicable Diseases Institute, Mazandaran University of Medical Sciences, Sari, Iran

**Keywords:** Blood pressure, Oral contraceptive, Systole, Diastole

## Abstract

**Background:**

The association between oral contraceptives (OCP) and hypertension has been reported in the literature with controversial results. According to the growing use of OCPs among women in Iran, this study aims to investigate the association between the duration of the OCP consumption and risk of hypertension among Iranian women.

**Methods:**

In the current study, the data collected during the enrolment phase of the Tabari cohort were analyzed. Of 6106 women recruited in the cohort, 133 pregnant women were excluded. Epidemiological variables were collected using pre-designed questionnaires as well as the health insurance evidences. In addition, blood pressure and anthropometric factors were measured based on the standard guidelines. Chi square and partial correlation tests as well as logistic regression models were applied for data analysis.

**Results:**

Frequency of oral contraceptive use among 35–70 year-old women in Tabari cohort study (TCS) was 42.2% (2520/5973). Hypertension was observed among 25% (1793/5973) of them. The adjusted odds ratio for OCP use was 1.23 (95% confidence interval: 1.08, 1.40, *p* = 0.002). The corresponding odds ratios for 61–120 months and more than 120 months OCP use were 1.39 (1.12,1.73) and 1.47 (1.16,1.87) respectively.

**Conclusions:**

Oral contraceptives especially in long term use can be associated with hypertension.

## Background

Non-communicable disease such as cardiovascular problems are main causes of death worldwide responsible for approximately half of the burden of diseases especially in low-middle income countries [1, 2]. Hypertension is the most common cause of the cardiovascular diseases as well as chronic kidney disorders and heart failure in worldwide. Hypertension is also responsible for at least 45% of the cardiovascular related mortality and 51% of deaths following stroke. About one billion adults are suffering from hypertension in the world and it is predicted to be increased to 1.5 billion until 2025. Many hypertensive cases remain undiagnosed without treatment. Therefore, sudden deaths and early mortality due to the consequences of hypertension are considerable [3–5]. The role of hypertension in mortality among women is higher than that of the other preventable cardiovascular risk factors [6].

Results of a meta-analysis reported the prevalence of hypertension among the Iranian population aged over 15 as of 20.4% (16.5, 24.2) [7]. The prevalence of this disorder among the Tabari cohort population was estimated as of 22.2% (2281/10,255) for all population, 24.9% (1523/6106) for women and 18.3% (758/4149) for men [8]. Another meta-analysis reported that Iranian women had hypertension about 1.3 % higher than Iranian men. The latter study, suggested the OCP use as one of the reasons for higher rates of hypertension among women [9].

Several evidences have introduced OCPs as one of the probable risk factors of the hypertension [6, 10, 11]. Kim and Park found that women used OCP more than 24 months, had 1.96 folds higher risk of hypertension compared to those never used OCP [6]. OCP use has getting increased worldwide especially in the recent years [12]. During the foundation of the family planning council from 1967, the use of oral contraceptives was initiated in Iran [13]. Contraceptive methods have significant role in reducing the problems following unintended pregnancy and abortion [14].

Among different methods of contraception, OCP use is more common because of various factors such as reversibility, lower side effects, high affectivity and also its role in reducing risk of dysmenorrhea, endometrial and ovarian cancers and ovarian cysts [15].

On the other hand, oral contraceptives may increase the risk of different disorders such as obesity, vomiting/nausea, vertigo, anxiety and depression [16, 17]. Due to the wide use of the OCPs among women during the fertility age, the attributed risk of these drugs is expected to be considerable. In the study conducted by Barikani and Saeedi among 328 Iranian women over 30, prevalence of hypertension was reported as of 32%, 8.8% of them experienced OCP use for 9.8 ± 6.1 years. However, they did not find any significant correlation between OCP use and hypertension [18]. In addition, oral contraceptives are widely used in Iran so that 24% of women in the capital city Tehran reported history of modern methods of contraception including OCPs [19].

As mentioned above, hypertension plays a critical role in the pathogenesis of non-communicable diseases. Although OCP is considered as one of the probable risk factors of the hypertension, controversies in the available literature due to various factors such as sample characteristics and study designs needs more investigations in different populations. The present study aims to investigate the association between OCP use and hypertension using the dose-response effect of oral contraceptives in the Tabari cohort population.

## Methods

The present study is a cross sectional (descriptive-analytic) research. Required information were obtained from the registered data of the enrolment phase of the Tabari population-based cohort. This cohort is a part of national cohort titled “Prospective Epidemiological Research Studies in IrAN (PERSIAN)” [20, 21]. More details of the cohort has been explained elsewhere [8].

The enrolment phase of the TABARI cohort has been established between 2015 and 2017. The study samples were selected by census method. Of 10,255 samples from the urban and mountainous areas of Sari district (capital of Mazandaran province in north of Iran), 6016 were women aged between 35 and 70 years. Some trained health volunteers in the urban areas as well as health staffs in the mountainous areas invited these participants based on the lists extracted from the health records.

The information were collected using standard questionnaires [8, 20, 21], physical examination and blood sampling. Before the study, the cohort staff of the Tabari study were trained and achieved the relevant qualification.

Some of the variables of the standard questionnaire required for the present study were age, height, weight, waist circumference, socioeconomic status, residence area, educational level, number of the previous pregnancies, menopausal status, familial history of hypertension, breast feeding duration, OCP use and its duration, history of hypertension and diabetes mellitus and using antihypertensive/anti-diabetic drugs, systolic/diastolic blood pressure, blood sugar, TG, TC, HDL and MET (metabolic equivalent of task).

OCP use was defined as at least one month consumption of the oral contraceptives during the fertility period until the questioning time in the enrolment phase of the cohort. Those reporting a history of the OCP use were also asked regarding the duration of the OCP consumption.

The participants’ blood pressure as well as anthropometric variables were measured according to PERSIAN cohort protocol. Blood samples were collected after 12 h fasting. Fasting blood glucose, TG and HDL were measured using Auto Analyzer, BT 1500 (Biotechnica, Italy). CBC was also measured using Alpha cell counter (Nihon Kohden, Tokyo, Japan).

Hypertension was defined when systolic blood pressure was ≥ 140 mmHg or diastolic pressure was ≥ 90 mmHg or participants reported previous history of hypertension. Physical activity of the participants was measured using a standard questionnaire calculating a MET score as a unit for activity.

SPSS version 24 was used for statistical analyses. All data were described as percent frequency, mean and standard deviation. Comparing the grouping variables between the participants with and without OCP use was performed using Chi square test. Independent T test was applied for comparison of the OCP use duration between these two groups. The association between hypertension and OCP use was assessed using logistic regression models. The adjusted models were run by stepwise method in seven models. To investigate the role of the OCP use duration, 1–11 months, 12–24 months, 25–60 months, 60–120 months and more than 120 months use were compared with “never use” group. Mean arterial pressure (MAP) was calculated using (2*DBP + SBP)/3 formula [22]. Spearman correlation and partial correlation coefficients were estimated to assess the crude and adjusted correlation between blood pressure and the duration of the OCP use. Due to the high correlation coefficient between the number of pregnancies and duration of breastfeeding, just the “number of pregnancies” variable was entered into the multivariate models.

## Results

Of 6106 women registered in the Tabari cohort, 133 pregnant women were excluded. Of the 5973 remaining study subjects, 17.93 (25%) had hypertension. Mean systolic, diastolic and MAP (mean arterial pressure) of the study subjects were 73.0 ± 7.7, 114.2 ± 13.7 and 86.8 ± 9.2 respectively.

History of OCP use was observed in 42.2% (2520/5973) of the present cohort population. Moreover, frequency of using oral contraceptives for 1–11 months, 12–24 months, 61–120 months and more than 120 months were 239 (4%), 640 (10.7%), 655 (11%), 549 (9.2%) and 437 (7.3%) respectively. Mean (SD) of OCP use among 2520 pregnant women reporting consumption of these drugs was 69.9 (65.8).

The epidemiological characteristics of the participants with and without OCP use has been shown in Table 1. Women with history of OCP use compare to those without had higher rate of obesity (45.5% vs. 40.4% respectively, *p* < 0.001), abdominal obesity (74.2 % vs. 66.5% respectively, *p* < 0.001) and diabetes mellitus (20.8% vs. 16.4% respectively, *p* < 0.001).

**Table 1:** Epidemiological characteristics of female participants of TCS

Variables (epidemiological characteristics)		Oral contraceptive use		*p *value (Chi square or Independent *T* test)
		Yes (n = 2520 )	No (n = 3453)	
Age group	35–39	13.0	19.2	< 0.001
	40–49	36.7	34.5	
	50–59	33.2	28.9	
	60–70	17.1	17.4	
BMI	< 25	13.8	20.0	< 0.001
	25-29.9	40.6	39.6	
	≥ 30	45.5	40.4	
WC	< 88	25.8	33.5	< 0.001
	≥ 88	74.2	66.5	
Social economic level	1	22.6	23.1	0.011
	2	21.2	20.1	
	3	21.2	19.6	
	4	19.4	18.2	
	5	15.6	18.9	
residence area	Urban	63.5	69.0	< 0.001
	Mountainous	36.5	31.0	
Educational level	University/college	12.6	20.2	< 0.001
	9–12 years in school	24.2	26.7	
	6–8 years in school	11.4	9.2	
	1–5 years in school	31.8	24.2	
	No schooling	20.0	19.7	
Parity	< 2	3.0	13.6	< 0.001
	2–3	45.1	43.1	
	4–5	31.7	20.2	
	> 5	20.2	23.1	
Menopause	No	52.1	56.8	< 0.001
	Yes	47.9	43.2	
HDL	≥ 50	60.0	58.4	0.230
	< 50	40.0	41.6	
TG	< 150	61.0	62.2	0.314
	≥ 150	39.0	37.8	
TC	< 200	59.6	61.5	0.148
	≥ 200	40.4	38.5	
Met	<Median	47.3	54.0	< 0.001
	≥Median	52.7	46.0	
Diabetes	No	79.2	83.6	< 0.001
	Yes	20.8	16.4	
Family history of hypertension	No	41.9	42.8	0.482
	Yes	58.1	57.2	
Breastfeeding, mean ± SD; month		66.2 ± 39.4	59.9 ± 41.7	< 0.001

Frequency of hypertension among OCP users was higher than that among women without history of OCP use (28.5% vs. 22.4% respectively, *p* < 0.001; OR = 1.38, 05% CI 1.22, 1.51). The crude and adjusted ORs based on different models has been presented in Table 2. The odds ratio for hypertension controlling for age was estimated as of 1.33 (1.18, 1.51). Corresponding figure adjusting for age, BMI, waist circumference, parity and co-morbidity with diabetes mellitus was 1.23 (1.08, 1.40, *p* = 0.002).

**Table 2:** Crude and adjusted associations (logistic regression) between hypertensive and oral contraceptive use

Variables	Model	Oral contraceptive use; OR (95% CI)		
		No	Yes	*p* value
Has hypertensive	Crud	Ref	1.38 (1.22–1.55)	< 0.001
	Adjusted for age	Ref	1.33 (1.18–1.51)	< 0.001
	Adjusted for Social economic, Education level, area resident	Ref	1.32 (1.17–1.49)	< 0.001
	Adjusted for BMI, WC, Met	Ref	1.30 (1.16–1.47)	< 0.001
	Adjusted for TG, TC, HDL-C, DM	Ref	1.32 (1.17–1.49)	< 0.001
	Adjusted for pregnancy number, Menopause	Ref	1.28 (1.13–1.45	< 0.011
	Adjusted for age, BMI, WC, pregnancy number, DM	Ref	1.23 (1.08–1.40)	0.002

Frequencies of hypertension among women without history of OCP use, OCP use for 1–11 months, 12–24 months, 25–60 months, 61–120 months and more than 120 months were 22.4%, 24.7%, 28.1%, 28.4%, 30.1 and 29.3% respectively (*p* < 0.001). Considering the OCP non-users as reference group, the adjusted odds ratios for 61–120 months and more than 120 months OCP use were estimated as of 1.39 (1.12, 1.73) and 1.47 (1.16, 1.87) respectively (Table 3).

**Table 3:** Crude and adjusted associations (logistic regression) between hypertension and duration of oral contraceptive use

Variables	Model	Duration of oral contraceptive use (month); OR (95% CI)					
		Never	1–11	12–24	25–60	61–120	> 120
Hypertension	Crude	Ref	1.13 (0.83–1.54)	1.35 (1.12–1.63)	1.37 (1.14–1.65)	1.48 (1.22–1.81)	1.43 (1.15–1.78)
	Adjusted age, BMI, WC, pregnancy number, DM	Ref	1.28 (0.92–1.79)	1.05 (0.86–1.30)	1.15 (0.93–1.40)	1.39 (1.12–1.73)	1.47 (1.16–1.87)

Table 4 shows that mean DBP, SBP and MAP were higher among women with OCP use compared to those without (*p* < 0.001). According to the results of partial correlation and controlling the effect of potential confounders, positive correlations were observed between the duration of the OCP use and the mean blood pressures.

**Table 4:** Crude and adjusted correlation coefficients between DBP, SBP and MAP with duration of oral contraceptive use

Variables	oral contraceptive use, Mean and 95% confidence interval		Correlation with duration of oral contraceptive use			
	No	Yes	Pearson correlation coefficient		Partial correlation coefficient*	
			r	*p* value	r	*p* value
DBP	72.6 (72.4–72.9)	73.6 (73.3–73.9)	0.07	< 0.001	0.05	< 0.001
SBP	113.3 (112.8–113.7)	115.5 (115.0–116.1)	0.07	< 0.001	0.05	< 0.001
MAP	86.2 (85.9–86.5)	87.6 (87.2–87.9)	0.07	< 0.001	0.06	< 0.001

*Adjusted by age, BMI, WC, pregnancy number, DM, Met

Also, among the 2520 study participants with history of OCP use, prevalence of Obesity, overweight and normal BMI was 45.5%, 40.6 and 13.8% respectively. Of them, 74.2% had high waist circumference (≥ 88 cm) (Table 1).

Prevalence of hypertension in women with obesity, overweight and normal BMI was 30.6%, 23 and 15.8% respectively (*p* < 0.001). In addition, prevalence of hypertension in women with abdominal obesity was significantly higher than that among women with normal waist circumference (29.5% vs. 14.6% respectively, *p* < 0.001).

## Discussion

The present study investigated the association between OCP use and hypertension among the TABARI cohort population. Our results showed that after adjustment of the effect of potential confounders, prevalence of hypertension among women with history of OCP use was 23% higher than that among those without OCP use. In addition, women used OCP for 61–120 months as well as those with OCP use more than 120 months had significantly 39 and 47% higher chance of developing hypertension compared to those without experience of OCP use. Moreover, duration of the OCP use was directly correlated with mean systolic, diastolic and arterial pressures.

Park et al. investigated the association between OCP use and hypertension among South Korean women aged 35–55 in 2013. Although using oral contraceptives for more than 24 months caused approximately two folds increase in the risk of developing hypertension, they did not find any significant risk of disease for less than two years OCP use [6]. Such dose response effect of OCP was similar to that observed in the current study.

According to the results of a meta-analysis conducted in 2017 by Liu et al., highest doses of OCP compared to the lowest doses caused approximately 50% higher risk of developing hypertension. They also showed the corresponding relative risks for Asian, North American, developed and developing countries as of 2.12 (1.15, 3.91), 1.32 (1.10, 1.59), 1.26 (1.09, 1.45) and 2.13 (1.06, 4.27) respectively. That meta-analysis showed 13% increase in the risk of hypertension per each 5 years higher OCP use [11]. These evidences are in parallel with the findings of our study.

According to the Wang study results, Chinese women with OCP use had approximately 40 % higher chance of developing hypertension compared to those without OCP use [22].

Another study in Chinese women showed the significant role of cumulative use of oral contraceptives so that using OCP more than 15 years caused about 50% higher risk of developing hypertension [10].

In contrast to the above results, OCP use did not have any significant effect on developing hypertension among women living in the other parts of Iran [18]. In addition, Farahmand et al. found that OCP use less than 36 months did not have significant association with cardiometabolic parameters [23]. There is no evidence of the effect of geographical areas and ethnicity on the association between OCP use and hypertension. Therefore, the observed differences between the results of the current study and the similar researches in other parts of Iran might be due to the other factors such as methodology and the characteristics of the study subjects. Similarly, OCP use and its duration did not play any role in developing hypertension among Australian women [24].

The most of the recent literatures have provided significant evidences regarding the positive effect of OCP use on developing hypertension. Ribeiro et al. reported the role of the estrogen component of the oral contraceptive pills on activation of the rennin angiotensin system leading to elevated blood pressure even in the lower doses [25]. Similarly, Takao Saruta reported an increased level of plasma rennin in the hypertensive patients using oral contraceptives [26]. However, some other studies did not agree with this mechanism. Other mechanisms such as the effect of progesterone, fluid retention by estrogen, renal abnormal function due to sodium-lithium countertransport and intra-renal vascular lesions [12]. It should be noted that the exact mechanism is necessary to be investigated by further studies. Such effect can be stronger in combination with other risk factors such as age, family history of hypertension, renal diseases and obesity at least 6 months after beginning the OCP use. Usually antihypertensive treatment is needed especially during the mild and reversible hypertensions without adverse consequences [6].

One of the strengths of the present study is the observed highly force of association indicating higher chance of hypertension. The second strength of our study is the observed dose-response association between OCP use and hypertension which is in keeping with the Hill criteria for causal association [27]. Although cross sectional studies have no enough power to prove the causal relationships because of the lack of temporal time relationships between the exposure and outcome, such evidence can be in favor of the role of OCP use in having more chance of hypertension among women. In addition, the repeatability of our results with the most similar evidences which is of great importance in the epidemiology and causal inference.

One of the main limitations of our study is lack of enough evidence regarding the temporal relationship between the exposure (OCP) and outcome (hypertension) of interest. The data supporting the results have been obtained cross-sectional from the first phase of the TCS. Therefore re-call bias due to remembering the exact time of OCP use and the first episode of the hypertension is expected. However, it seems that re-call bias will be low in use of methods such as contraception. Moreover, the health records of the Iranian health centers used as the main source of the baseline information is reliable leading to the low risk of systematic error.

## Conclusions

Our study showed higher rates of hypertension among women with the history of OCP use. We also found a considerable direct effect of the duration of contraception with pills. Future longitudinal studies focusing on temporal relationship between OCP use and hypertension, beside the observed dose-response effects, can provide more credible evidences to introduce oral contraceptives as independent risk factors for developing hypertension.

## Data Availability

The datasets used and/or analysed during the current study are available from the corresponding author on reasonable request.

## Abbreviations

TCS: Tabari Cohort Study
OCP: oral contraceptive
DM: diabetes mellitus
BMI: body mass index
WC: waist circumference
MET: metabolic equivalent of task
TG: triglycerides
TC: total cholesterol
HDL-C: high density lipoprotein
DBP: diastolic blood pressure
SBP: systolic blood pressure
MAP: mean arterial pressure
CBC: complete blood count
OR: odds ratio
